# STAFNet: an adaptive multi-feature learning network via spatiotemporal fusion for EEG-based emotion recognition

**DOI:** 10.3389/fnins.2024.1519970

**Published:** 2024-12-10

**Authors:** Fo Hu, Kailun He, Mengyuan Qian, Xiaofeng Liu, Zukang Qiao, Lekai Zhang, Junlong Xiong

**Affiliations:** ^1^College of Information Engineering, Zhejiang University of Technology, Hangzhou, China; ^2^Department of Tuina, The First Affiliated Hospital of Zhejiang Chinese Medical University (Zhejiang Provincial Hospital of Chinese Medicine), Hangzhou, China; ^3^The School of Design and Architecture, Zhejiang University of Technology, Hangzhou, China

**Keywords:** EEG, emotion recognition, deep learning, spatiotemporal fusion, adaptive adjacency matrix

## Abstract

**Introduction:**

Emotion recognition using electroencephalography (EEG) is a key aspect of brain-computer interface research. Achieving precision requires effectively extracting and integrating both spatial and temporal features. However, many studies focus on a single dimension, neglecting the interplay and complementarity of multi-feature information, and the importance of fully integrating spatial and temporal dynamics to enhance performance.

**Methods:**

We propose the Spatiotemporal Adaptive Fusion Network (STAFNet), a novel framework combining adaptive graph convolution and temporal transformers to enhance the accuracy and robustness of EEG-based emotion recognition. The model includes an adaptive graph convolutional module to capture brain connectivity patterns through spatial dynamic evolution and a multi-structured transformer fusion module to integrate latent correlations between spatial and temporal features for emotion classification.

**Results:**

Extensive experiments were conducted on the SEED and SEED-IV datasets to evaluate the performance of STAFNet. The model achieved accuracies of 97.89% and 93.64%, respectively, outperforming state-of-the-art methods. Interpretability analyses, including confusion matrices and t-SNE visualizations, were employed to examine the influence of different emotions on the model's recognition performance. Furthermore, an investigation of varying GCN layer depths demonstrated that STAFNet effectively mitigates the over-smoothing issue in deeper GCN architectures.

**Discussion:**

In summary, the findings validate the effectiveness of STAFNet in EEG-based emotion recognition. The results emphasize the critical role of spatiotemporal feature extraction and introduce an innovative framework for feature fusion, advancing the state of the art in emotion recognition.

## 1 Introduction

Emotion recognition is an essential component of daily life, playing an increasingly pivotal role in both interpersonal communication and cognitive decision-making. Consequently, developing more intelligent emotion recognition algorithms is crucial for enhancing both accuracy and efficiency (Chen et al., 2021). Emotion recognition data can be broadly categorized into two types: non-physiological signals [e.g., facial expressions (Huang et al., 2019), speech (Wang et al., 2022)] and physiological signals [e.g., electrocardiograms (ECG) (Meneses Alarcão and Fonseca, 2017), electrodermal activity (EDA) (Veeranki et al., 2024a), and electroencephalograms (EEG) (Veeranki et al., 2024b)]. Although non-physiological signals provide intuitive insights into emotional states, they are subject to manipulation, as individuals may intentionally conceal their true emotions. In contrast, physiological signals offer a more objective reflection of an individual's authentic emotional state (Li et al., 2020). Among these, EEG signals are particularly noteworthy for their ability to capture emotional stimuli directly affecting the central nervous system (Berboth and Morawetz, 2021). As a result, EEG-based emotion recognition is anticipated to attract increasing research attention.

The inherent instability of EEG signals and the complexity of brain structure make it particularly challenging to analyze and extract latent features for distinguishing between emotional states. Current feature learning methods can be broadly classified into traditional machine learning and deep learning approaches. Traditional machine learning methods require manual extraction of shallow features, such as Hjorth parameters, higher-order crossings (HOC), power spectral density (PSD), and differential entropy (DE) (Yan et al., 2022; Jenke et al., 2014; Duan et al., 2013). However, these methods heavily depend on expert knowledge, which may limit a holistic understanding of the intricate emotion-related EEG features. To address these limitations, a growing body of research has turned to deep learning techniques for feature extraction, which has significantly enhanced the performance of emotion recognition systems (Ngai et al., 2021; Zuo et al., 2024b). Currently, deep learning approaches mainly focus on extracting features from the temporal and spatial dimensions. For temporal feature extraction, recurrent neural networks (RNNs) have been employed to capture the dynamic temporal patterns in EEG signals (Wei et al., 2020; Wu et al., 2024; Hu et al., 2024b). Chen et al. (2019) introduced a hierarchical bidirectional gated recurrent unit (BiGRU) network to mitigate the effects of long-term non-stationarity in EEG signals by focusing on temporal features. Similarly, Algarni et al. (2022) utilized a stacked bidirectional long short-term memory (Bi-LSTM) network to generate emotion-related feature sequences in chronological order, achieving an accuracy of 96.87% on the DEAP dataset. While these studies effectively highlight the importance of temporal information, they overlook the topological structure of the brain, which plays a crucial role in understanding brain connectivity in emotional recognition. In terms of spatial feature extraction, convolutional neural networks (CNNs) had become the preferred choice for this domain (Rahman et al., 2021; Bagherzadeh et al., 2022). CNNs possess robust feature extraction capabilities and are adept at effectively processing continuous dense feature maps, thereby demonstrating exceptional performance in handling spatial relationships. However, the sparse spatial structure of EEG channels limits CNNs' ability to fully explore the spatial relationships between channels. To overcome this limitation, graph convolutional networks (GCNs) have been increasingly adopted to model the adjacency relationships between EEG channels. By constructing topological representations of the brain to extract deep spatial features, GCNs have shown significant promise in emotion recognition tasks (Chang et al., 2023; Zong et al., 2024). For instance, Wang et al. (2019) proposed a phase-locking value (PLV)-based graph convolutional neural network (P-GCNN), which constructs an adjacency matrix by calculating the phase synchronization between EEG channels using PLV. This method addresses the discrepancy between the spatial, physical locations of EEG channels and their functional connections. Nevertheless, while static graph construction based on functional connectivity helps to capture stable spatial patterns in EEG signals, its reliance on prior knowledge makes it difficult to dynamically capture the evolving dependencies between nodes driven by emotional fluctuations. Thus, developing a model that effectively integrates both the temporal and spatial features of EEG signals is essential for achieving a more comprehensive and nuanced analysis of emotion recognition.

Effectively capturing the consistency and complementarity of multi-feature information in emotional semantics is a critical area of research in emotion recognition. Consistency refers to the shared semantic information across different features, while complementarity highlights the distinct semantic information unique to each feature. Multi-feature fusion methods are generally divided into three categories: feature-level fusion (Zhang et al., 2024), decision-level fusion (Pu et al., 2023), and model-level fusion (Islam et al., 2024). Feature-level fusion involves combining various features into a single feature vector to form a comprehensive representation. For example, Tao et al. (2024) proposed an attention-based dual-scale fusion convolutional neural network (ADFCNN) that integrates spectral and spatial information from multi-scale EEG data using a concatenation fusion strategy. However, ADFCNN directly merges features from different sources, potentially overlooking essential spatial information within individual features and the temporal synchronization between them. Decision-level fusion, on the other hand, combines multiple predictions using algebraic rules. Dar et al. (2020) utilized CNNs and long short-term memory networks (LSTMs) to separately process EEG signals, followed by a majority voting mechanism to generate the final classification. However, since data is processed independently by different networks, this method limits the transfer of complementary information between features. Model-level fusion aims to foster interactions between different feature domains, allowing the model to uncover correlations and fully exploit the complementary nature of multiple features. Huang et al. (2023) introduced a model called CNN-DSC-BiLSTM-Attention (CDBA), which employs a multi-branch architecture to extract diverse features from EEG signals and uses a self-attention mechanism to assign feature weights for emotion classification. While the self-attention mechanism effectively captures internal dependencies within sequences, it has limitations when it comes to integrating features from various information sources. This shortcoming arises because self-attention primarily focuses on the relevance of local or internal features, often neglecting the complex interactions between multiple feature domains (Hu et al., 2024a). Thus, a comprehensive understanding of the intrinsic connections between emotional expression and spatiotemporal information, along with a precise modeling of the interactions between different features, is crucial for improving the model's capacity to recognize and track emotional patterns effectively.

Given the challenges outlined above, this paper introduces a novel network named the Spatiotemporal Adaptive Fusion Network (STAFNet), which integrates adaptive graph convolution and temporal transformers to enhance the accuracy and robustness of EEG-based emotion recognition. STAFNet is designed to fully exploit both the spatial topological structure and temporal dynamics of EEG signals. The Temporal Self-Transformer Representation Module (TSRM) emphasizes the most informative EEG segments within each channel, enabling the extraction of global contextual temporal information. Simultaneously, the Adaptive Graph Convolutional Module (AGCM) leverages an adaptive adjacency matrix to capture the dynamic patterns of brain activity, thus enabling the extraction of highly discriminative spatial features. Finally, the Multi-Structured Transformer Fusion Module (MSTFM) learns and integrates potential correlations between temporal and spatial features, adaptively merging key features to further boost model performance. The effectiveness of the proposed STAFNet is demonstrated through performance comparisons with state-of-the-art (SOTA) methods and validated via ablation studies. The key innovations of this paper are as follows:

- We propose an AGCM to explore the spatial connections between brain channels. To capture the dynamic changes in brain network structure over time, this module adaptively updates the adjacency matrix during backpropagation, allowing it to reflect temporal variations in brain connectivity.
- We integrated an enhanced transformer into the MSTFM, which employs a novel attention mechanism to effectively fuse complementary spatiotemporal information from EEG signals. This allows the model to capture the intrinsic connections between emotional expression and spatiotemporal features, leading to a significant improvement in classification performance.
- STAFNet employs a dual-branch architecture to seamlessly integrate both temporal and spatial feature information from EEG signals. Experimental results demonstrate that STAFNet outperforms SOTA methods on the public SEED and SEED-IV datasets, showcasing its superior performance in EEG-based emotion recognition.

## 2 Materials and methods

### 2.1 Datasets

To evaluate the proposed model, we conducted EEG-based emotion recognition experiments using the Shanghai Jiao Tong University Emotion EEG Database (SEED) (Zheng and Lu, 2015) and its enhanced version, SEED-IV (Zheng et al., 2019). These datasets were employed to demonstrate the effectiveness and robustness of the STAFNet model.

**Figure 1:**
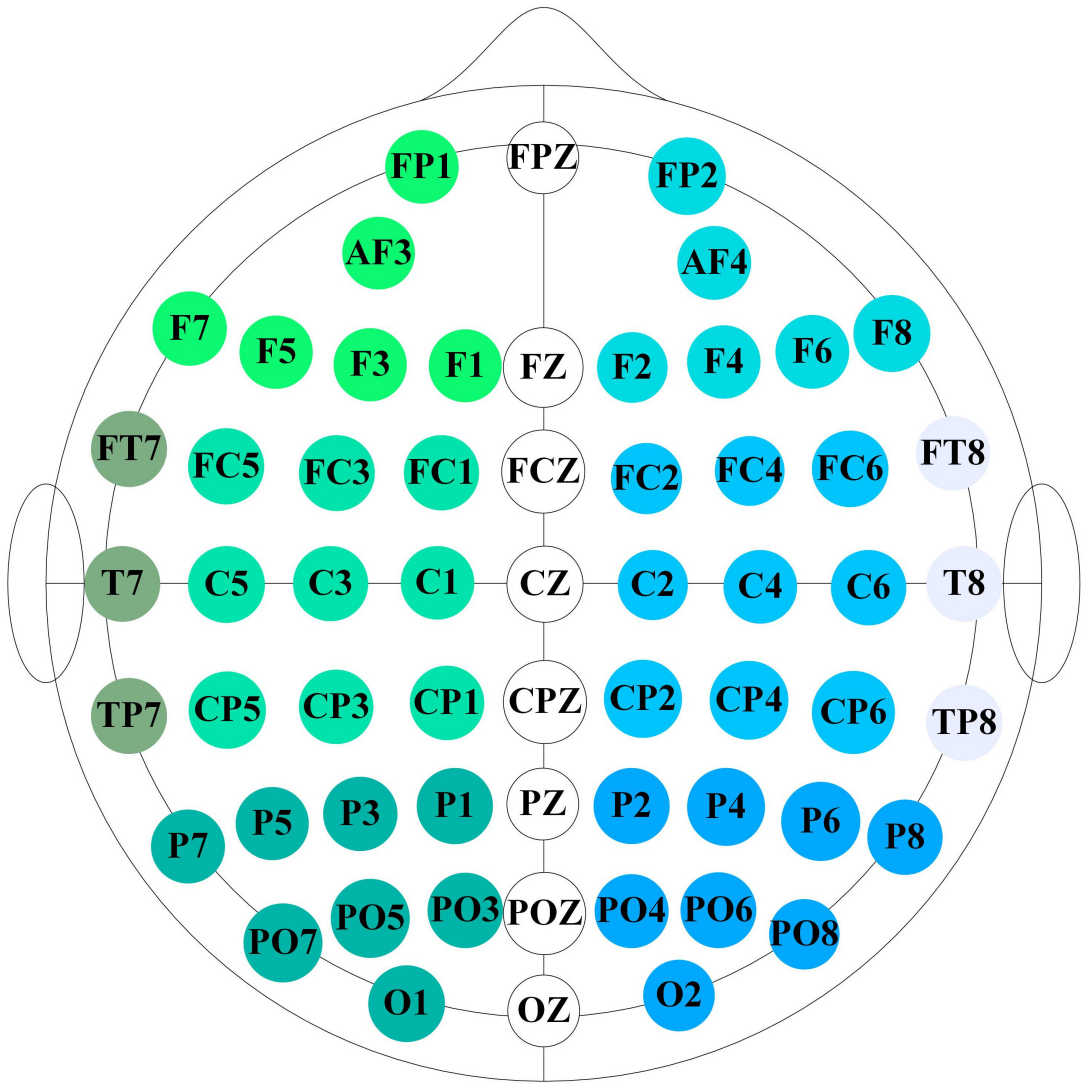
A schematic diagram of the 62 electrodes in the EEG cap used for the SEED and SEED-IV datasets. The diagram shows the approximate locations of each electrode on the brain.

The SEED dataset consists of EEG recordings from 15 participants (7 males and 8 females) while they watched 15 movie clips, each representing one of three emotions: positive, neutral, or negative. Each clip lasted approximately 4 minutes. The experiment was conducted in three separate sessions, with intervals between sessions, resulting in EEG data collected from all 15 participants across three sessions. EEG data were recorded using a 62-channel ESI NeuroScan system, with electrode placement following the international 10-20 system, as illustrated in Figure 1. In total, the SEED dataset contains 675 EEG samples (45 trials per participant for 15 subjects). For each participant, there are 15 samples corresponding to each emotional category.

The experimental process for the SEED-IV dataset is similar to that of the SEED dataset, but with a broader range of emotions and more movie clips. In SEED-IV, 72 movie clips were selected to elicit four emotions: happiness, sadness, fear, and neutrality, offering a wider emotional spectrum compared to SEED. Fifteen participants took part in the experiments, conducted at regular intervals, with each session consisting of 24 trials. EEG data were recorded for each participant using a 62-channel ESI NeuroScan system, following the international 10-20 electrode placement system. The SEED-IV dataset contains a total of 1,080 samples (72 trials per participant across 15 subjects), with each participant contributing 18 samples for each emotion type.

### 2.2 Preprocessing

In both the SEED and SEED-IV datasets, EEG signals were originally sampled at 1,000 Hz and then downsampled to 200 Hz. To ensure a fair comparison with existing studies (Zeng et al., 2022), we adopted the same preprocessing strategy. First, the EEG data from both datasets were segmented using non-overlapping sliding windows of 1-s duration to maintain temporal continuity and consistency. A 3rd-order Butterworth bandpass filter was then applied to the raw EEG data to retain the frequency bands relevant for emotion recognition while effectively suppressing high-frequency and low-frequency noise. Finally, Z-score normalization was applied to mitigate variability and address non-stationarity in the EEG signals.

### 2.3 Proposed methodology

**Figure 2:**
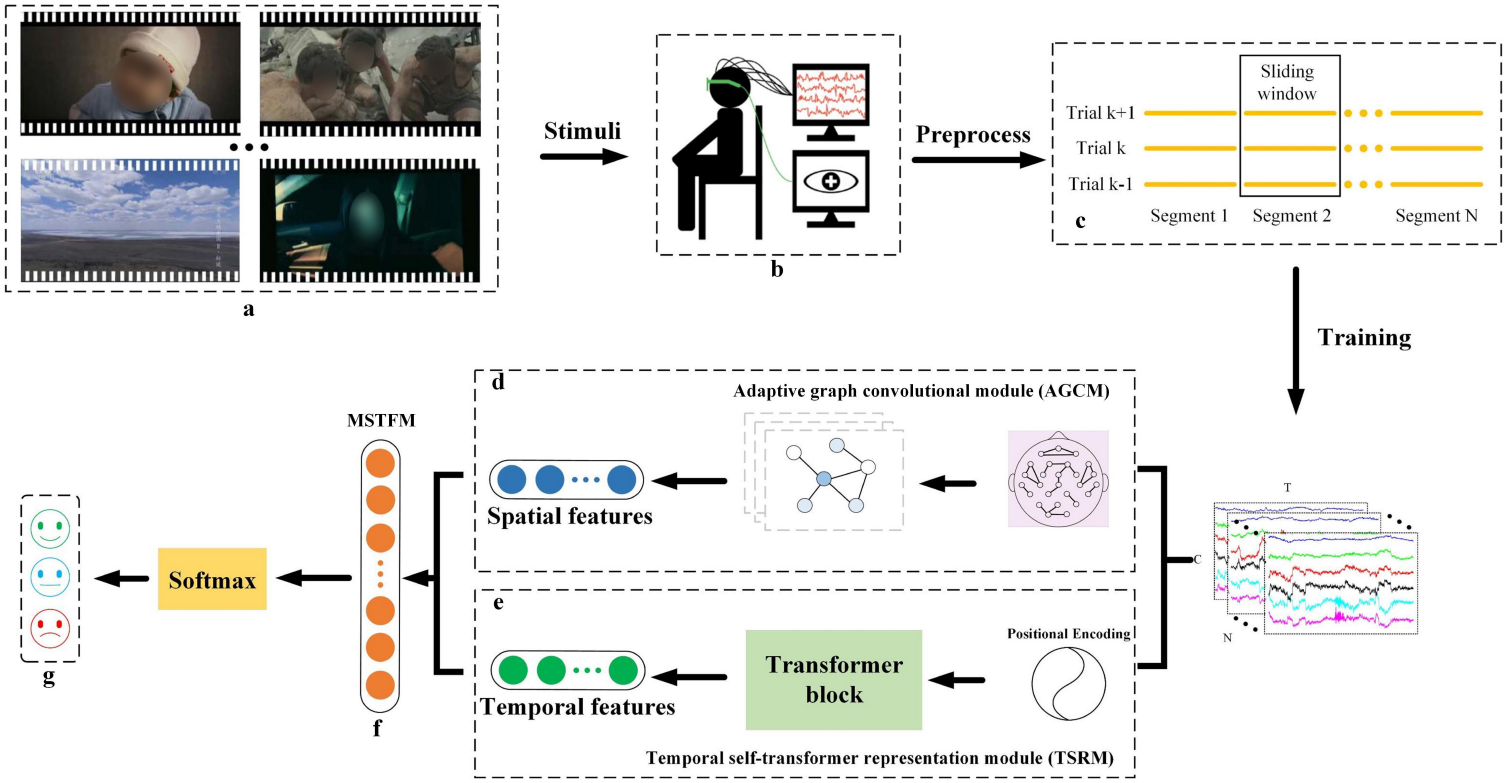
Overview of the STAFNet model architecture for EEG-based emotion recognition. **(A)** Emotional stimulus; **(B)** Acquisition of input data; **(C)** EEG signal slicing; **(D)** AGCM, which extracts the spatial features of the EEG signals; **(E)** TSRM, which extracts the temporal features of the EEG signals; **(F)** MSTFM, which fusion the spatial and temporal features; **(G)** the classification results.

Our proposed STAFNet model proficiently extracts and integrates spatial and temporal features from EEG signals, enabling accurate emotion recognition. STAFNet consists of four main functional components: AGCM, TSRM, MSTFM, and CM. The input to the model is represented as $X=\left[x_{1},\ldots ,x_{n}\right]\in {\mathbb{R}}^{N\times T\times C}$, where *n* denotes the *n*-th preprocessed EEG sample, *N* denotes the total number of samples, *T* denotes the sample length, and *C* denotes the number of EEG channels. In the entire framework, the preprocessed EEG signals are fed into the STAFNet model, with dimensions denoted as [*N, T, C*]. Figure 2 provides an overview of the STAFNet model's process for handling EEG data. First, the raw EEG signals are preprocessed and segmented to obtain the input representation *X*. Next, *X* are processed through the AGCM and TSRM to extract highly discriminative spatial and temporal features, respectively. Subsequently, the MSTFM is used to integrate the complementary information between spatial and temporal features, resulting in the fused features. Finally, the fused features are processed through the CM layer to obtain the final prediction results.

#### 2.3.1 Adaptive graph convolutional module

The dynamic connectivity patterns underlying emotional changes rely heavily on the spatial connections between electrodes. Thus, accurate connectivity estimation is crucial for understanding the interactions and information flow between different brain regions (Zuo et al., 2024a, 2023). We introduces an AGCM based on an adaptive adjacency matrix to capture spatial variation information. Specifically, we use a directed weighted graph *G* = (*V, A*), where *V* = {*v*_1_, *v*_2_, …, *v*_*n*_} denotes the set of vertices with *n* nodes, and the adjacency matrix *A* = (_*a*_*i,j*_)*n*×*n*_ describes the edge weights between nodes in *V*. Each element *a*_*i,j*_ denotes the coupling strength of the connection between node *i* and node *j*.

**Figure 3:**
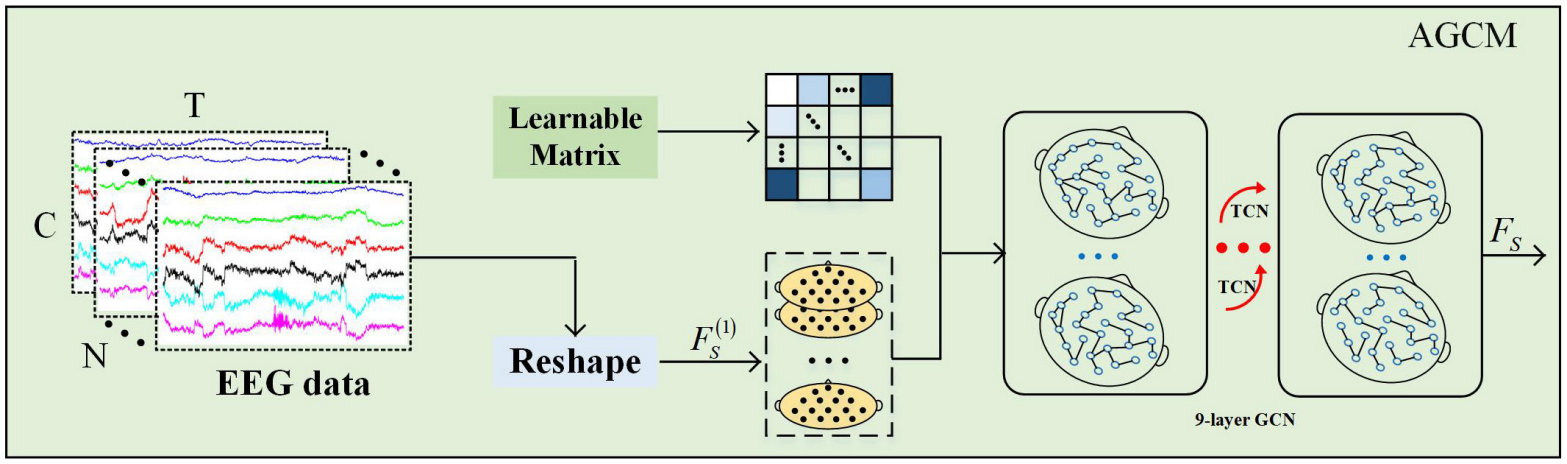
The AGCM.

To evaluate the potential relational variations between any two electrode channels, we propose a novel method for adaptively and dynamically learning the relationships between adjacent nodes, as illustrated in Figure 3. First, an adjacency matrix $A_{D}\in {\mathbb{R}}^{C\times C}$ is randomly initialized, where *C* denotes the number of channels. This adjacency matrix reflects the correlations between each pair of channels, accounting for both direction and intensity. Then, during model training, the weights of all channels in the adjacency matrix *A*_*D*_ are dynamically updated through a backpropagation mechanism, with the calculation formula as follows:


(1) $$\begin{array}{l} \{\begin{array}{l} Ã=W_{2}\delta \left(W_{1}A\right)\\ {Ã}_{D}=\sigma \left(Ã\right) \end{array} \end{array}$$

where $W_{1}\in {\mathbb{R}}^{\left(\frac{C\times C}{r}\right)\times \left(C\times C\right)}$ and $W_{2}\in {\mathbb{R}}^{\left(C\times C\right)\times \left(\frac{C\times C}{r}\right)}$ denote the weight matrices, δ(·) and σ(·) denote the Tanh and Relu function, and *r* is the reduction ratio. We introduce the Tanh function to model the directionality between different channels and employ an activation function Relu to enhance the coupling of significant channels while suppressing weaker channel connections, thereby obtaining an adaptive adjacency matrix Ã_*D*_. This approach enables the model to effectively handle varying emotional patterns and facilitates end-to-end learning.

To fully exploit the temporal information in the data, we incorporated a temporal convolution module and multiple residual connections on top of the dynamic graph convolution. This strategy not only enables the AGCM to capture local dependencies in the temporal dimension but also accurately captures the dynamic evolution characteristics of nodes in the spatial dimension. First, to capture spatial relationships, a transformation operation is applied to convert the preprocessed EEG data input *X* into $F_{S}^{\left(1\right)}\in {\mathbb{R}}^{N\times 1\times T\times C}$ to obtain the latent spatial features, where 1 denotes the initial feature dimension of AGCM. The update process for each layer of AGCM can be defined as follows:


(2) $$\begin{array}{l} F_{S}^{\left(l\right)}=\text{TCN}\left(\sigma \left({Ã}_{D}F_{S}^{\left(l-1\right)}W_{G}\right)\right)+F_{S}^{\left(l-1\right)},l\in \left[1,L\right] \end{array}$$

here, TCN(·) denotes the temporal convolution layer, *W*_*G*_ denotes the weights of the graph convolution layer, and $F_{S}^{\left(l-1\right)}$ denotes to the spatial features output from the previous layer. In this design, we employ a deep GCN design with *L* = 6 layers to explore latent dependencies between nodes in the EEG electrode channels, thereby extracting key spatial features $F_{S}\in {\mathbb{R}}^{N\times T\times C}$.

#### 2.3.2 Temporal self-transformer representation module

Different time points in EEG are interrelated, with each time point contributing differently to the emotion recognition task, making it crucial to analyze temporal features of EEG. To focus on more valuable temporal information, TSRM must effectively capture the global temporal dependencies of the EEG signal, assigning higher scores to the most relevant temporal information through a self-attention-based transformer mechanism.

**Figure 4:**
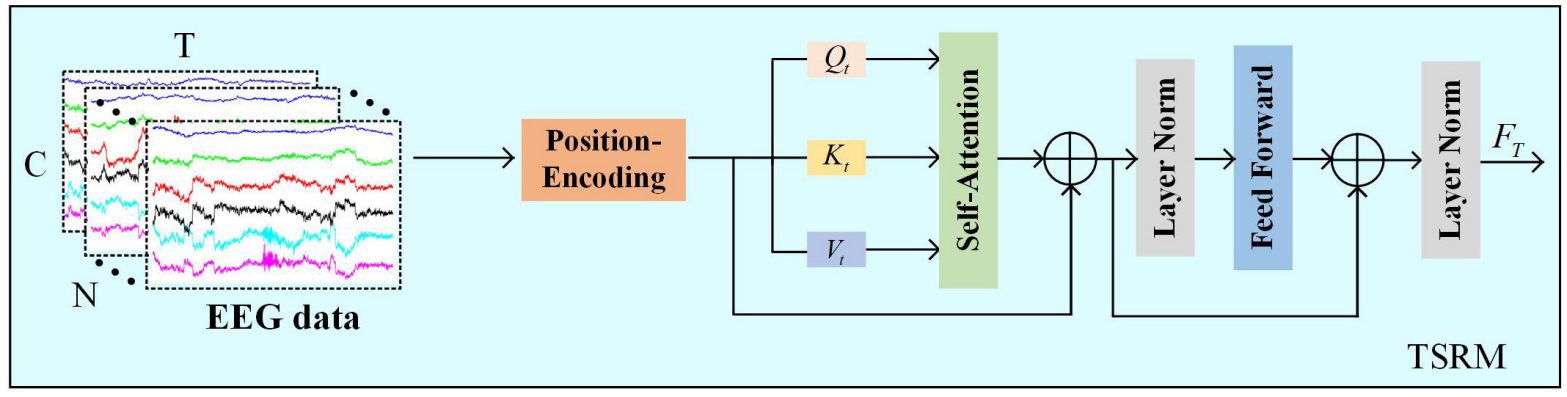
The TSRM.

As shown in Figure 4, TSRM primarily consists of positional encoding, self-attention mechanism, feed-forward layers, and regularization layers. Firstly, we use the preprocessed EEG data *X* as temporal features and introduce relative positional encoding (PE) to help the model capture the dependencies between different positions in the time series. Let the temporal positions be denoted as *pos* and the time points as *t*, the positional encoding is described as follows:


(3) $$\begin{array}{l} \{\begin{array}{l} PE_{\left(pos,2t\right)}=\sin\left(\frac{pos}{10000^{2t/d}}\right)\\ PE_{\left(pos,2t+1\right)}=\cos\left(\frac{pos}{10000^{2t/d}}\right) \end{array} \end{array}$$

In this context, *d* represents the dimension of the temporal vectors. To construct these temporal vectors, we employ sine functions to encode positional information for even time points, while cosine functions are utilized for odd time points.

We then add the positional encoding vectors *PE* to the feature vectors of the input sequence *X* to generate the final feature representation:


(4) $$\begin{array}{l} F_{PE}=X+PE \end{array}$$

here, *F*_*PE*_ denotes the feature map after relative positional encoding. Then, TSRM obtains the query vectors (*Q*_*t*_), key vectors (*K*_*t*_), and value vectors (*V*_*t*_) by multiplying the feature map with three different weight matrices. Subsequently, the dot product is computed between the query vectors *Q*_*t*_ and all key vectors *K*_*t*_, and adjusted by a scaling factor $\sqrt{d_{k}}$.

Next, the Softmax function is applied to normalize the adjusted dot product values, generating a score for each value. The computation process for the typical score matrix across all channels is as follows:


(5) $$\begin{array}{l} \left(Q_{t},K_{t},V_{t}\right)=\left(\left(F_{PE}W_{t}^{Q}\right),\left(F_{PE}W_{t}^{K}\right),\left(F_{PE}W_{t}^{V}\right)\right) \end{array}$$

(6) $$\begin{array}{l} \text{Attention}\left(Q_{t},K_{t}\right)=\text{Softmax}\left(\frac{Q_{t}K_{t}^{T}}{\sqrt{d_{k}}}\right) \end{array}$$

here, $W_{t}^{Q}\in {\mathbb{R}}^{C\times d}$, $W_{t}^{K}\in {\mathbb{R}}^{C\times d}$, $W_{t}^{V}\in {\mathbb{R}}^{C\times d}$ and denote the parameters of the linear transformations. The shape of the output matrix Attention(*Q*_*t*_, *K*_*t*_) is [*N, T, T*].

In the score matrix Attention(*Q*_*t*_, *K*_*t*_), values across all channels are aggregated with the available information to update the matrix. To mitigate the vanishing gradient problem, residual connections are incorporated. Furthermore, self-attention is integrated with a feed-forward network (FFN) consisting of two fully connected layers followed by a ReLU activation function. The process is delineated as follows:


(7) $$\begin{array}{l} V_{T}^{*}=\text{Attention}\left(Q_{T},K_{T}\right)V_{T} \end{array}$$

(8) $$\begin{array}{l} F_{res}=\text{LN}\left(V_{T}^{*}+F_{PE}\right) \end{array}$$

(9) $$\begin{array}{l} F_{T}=\text{LN}\left(F_{res}+\text{FFN}\left(F_{res}\right)\right)\in {\mathbb{R}}^{N\times T\times C} \end{array}$$

here, $V_{T}^{*}\in^{N\times T\times C}$ and $F_{res}\in {\mathbb{R}}^{N\times T\times C}$ denote the output features from the self-attention mechanism and the FFN, respectively. LN(·) denotes layer normalization, which is incorporated into the TSRM to reduce training time and enhance the model's generalization capability.

#### 2.3.3 Multi-structured transformer fusion module

**Figure 5:**
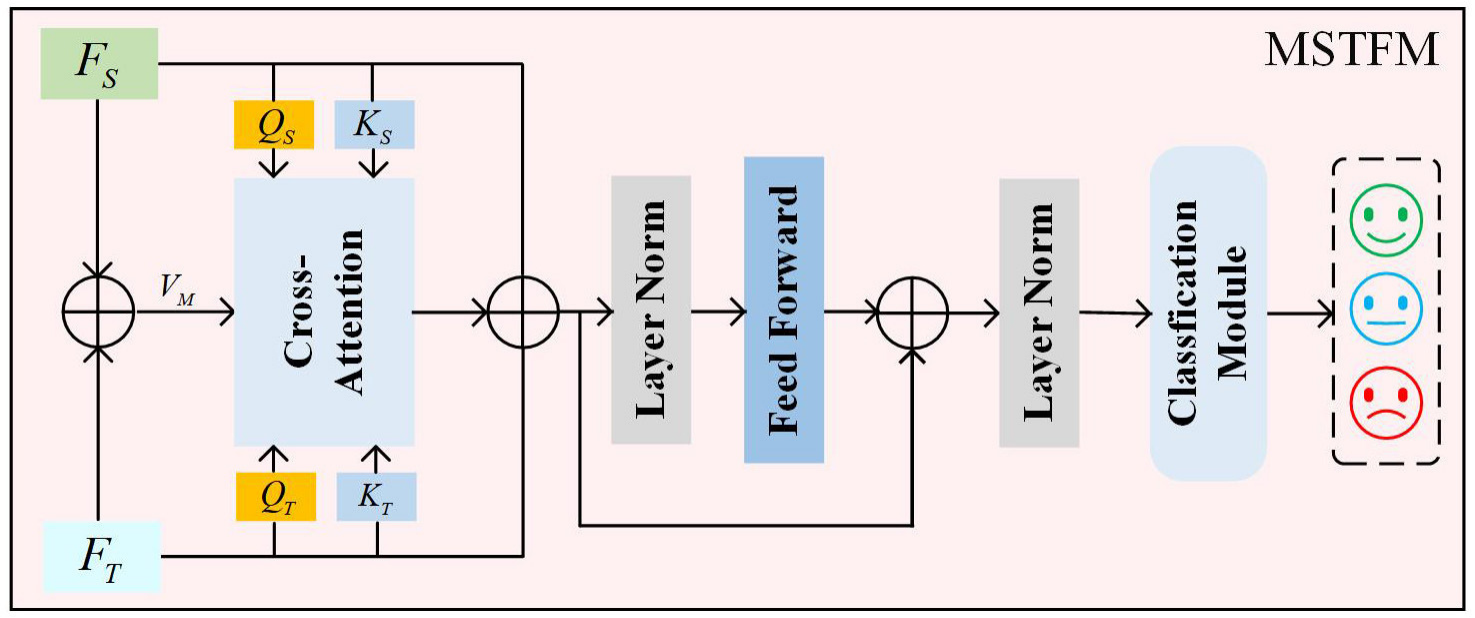
The MSTFM.

Through the aforementioned steps, we obtain spatial feature *F*_*S*_ and temporal feature *F*_*T*_. Cross-attention-based spatiotemporal feature fusion methods use features from one modality to guide the learning weights of features from another modality. However, this approach does not balance the importance between the two types of features. Traditional simple combination methods (e.g., weighted combination of two cross-attention blocks) may lead to data sparsity and require more computational resources. Therefore, we propose a novel cross-attention mechanism to leverage the complementary information between different modalities, enabling the model to extract more representative features, as illustrated in Figure 5.

First, to fully utilize the correlation and complementarity between different modality features, we introduce intermediate features to mitigate the discrepancies between these features. The intermediate features are obtained by a weighted summation of *F*_*S*_ and *F*_*T*_, as detailed in the following formula:


(10) $$\begin{array}{l} F_{M}=F_{T}⊕F_{S},F_{M}\in {\mathbb{R}}^{N\times T\times C} \end{array}$$

here, *F*_*M*_ denotes the intermediate state features, and ⊕ denotes the weighted summation operation. Through the module's weight learning, the attention weights for both features are guided by the intermediate state features, thereby uncovering shared semantic information between features and emphasizing the differences in their semantic information.

Next, the spatial features *F*_*S*_ and temporal features *F*_*T*_ are multiplied by different weight matrices to obtain their respective query vectors (*Q*_*S*_, *Q*_*T*_) and key vectors (*K*_*T*_, *K*_*S*_). Simultaneously, the intermediate state features *F*_*M*_ are multiplied by a weight matrix $W_{M}^{V}$ to obtain the value vectors *V*_*M*_. The specific formulas are as follows:


(11) $$\begin{array}{l} \left(Q_{S},K_{S},Q_{T},K_{T}\right)=\left(\left(F_{S}W_{S}^{Q}\right),\left(F_{S}W_{S}^{K}\right),\left(F_{T}W_{T}^{Q}\right),\left(F_{T}W_{T}^{K}\right)\right) \end{array}$$

(12) $$\begin{array}{l} V_{M}=F_{M}W_{M}^{V} \end{array}$$

here, $W_{S}^{Q}\in {\mathbb{R}}^{C\times d}$, $W_{S}^{K}\in {\mathbb{R}}^{C\times d}$, $W_{T}^{Q}\in {\mathbb{R}}^{C\times d}$, $W_{T}^{K}\in {\mathbb{R}}^{C\times d}$ and $W_{M}^{V}\in {\mathbb{R}}^{C\times d}$ represent the weight parameters for the linear transformations.

Then, based on the principles of the cross-attention mechanism, the query vectors (*Q*_*S*_, *Q*_*T*_) and key vectors (*K*_*S*_, *K*_*T*_) obtained from different features are used to compute two typical score matrices, as detailed in the following formulas:


(13) $$\begin{array}{l} \{\begin{array}{l} CA_{ST}\left(Q_{S},K_{T}\right)=\text{Softmax}\left(\frac{Q_{S}{\left(K_{T}\right)}^{T}}{\sqrt{d}}\right)\\ CA_{TS}\left(Q_{T},K_{S}\right)=\text{Softmax}\left(\frac{Q_{T}{\left(K_{S}\right)}^{T}}{\sqrt{d}}\right) \end{array} \end{array}$$

here, Softmax(·) denotes the Softmax activation function, and $\sqrt{d}$ represents the scaling factor. $CA_{ST}\left(Q_{S},K_{T}\right)\in {\mathbb{R}}^{N\times T\times T}$ measures the attention score of temporal features from the perspective of spatial features. $CA_{TS}\left(Q_{T},K_{S}\right)\in {\mathbb{R}}^{N\times T\times T}$ measures the attention score of spatial features from the perspective of temporal features.

Finally, the *CA*_*ST*_(*Q*_*S*_, *K*_*T*_) and *CA*_*TS*_(*Q*_*T*_, *K*_*S*_) score matrices are aggregated with the value vectors *V*_*M*_ to update the matrices. The proposed cross-attention mechanism integrates these two cross-attention matrices, providing a composite measure of the correlations between temporal and spatial features. The final result is defined as follows:


(14) $$\begin{array}{l} V_{M}^{*}=CA_{ST}\left(Q_{S},K_{T}\right)\times V_{M}\times CA_{TS}\left(Q_{T},K_{S}\right) \end{array}$$

(15) $$\begin{array}{l} F_{M}^{*}=\text{LN}\left(V_{M}^{*}+F_{S}+F_{T}\right) \end{array}$$

(16) $$\begin{array}{l} F_{MSTFM}=\text{LN}\left(F_{M}^{*}+\text{FFN}\left(F_{M}^{*}\right)\right) \end{array}$$

here, FFN(·) is a feedforward network implemented by a linear layer with an output dimension of $F_{M}^{*}$.

#### 2.3.4 Classification module

To further integrate the information in the fused result *F*_*MSTFM*_, which comprises temporal and spatial features, we employ four linear layers as a classification module to derive the final high-level features *F*_*CM*_ by inputting *F*_*MSTFM*_ into the CM. The linear layers have parameter dimensions of (128, 64, 32, *classnum*) in sequence, where *classnum* denotes the number of sentiment classes in the dataset. Finally, the output of the last linear layer is passed through a softmax activation function to obtain the predicted labels ${Ŷ}_{o}\in {\mathbb{R}}^{N}$:


(17) $$\begin{array}{l} F_{CM}=LN{\left[F_{MSTFM}\right]}_{4} \end{array}$$

(18) $$\begin{array}{l} {Ŷ}_{o}=\text{Softmax}\left(F_{CM}\right) \end{array}$$

here, *LN*[·]_4_ denotes the four linear operations.

The proposed STAFNet model employs the cross-entropy loss function in conjunction with *L*_2_ regularization to quantify the discrepancy between the true labels *Y*_*i*_ and predicted labels Ŷ_*o*_. The adjacency matrix is updated via the backpropagation mechanism. The process for updating the model parameters can be articulated as follows:


(19) $$\begin{array}{l} Loss=\text{crossentropy}\left(Y_{i},{Ŷ}_{o}\right)+\alpha ||\Theta |{|}_{2} \end{array}$$

here, Θ denotes all the parameters in the model training process, α denotes the weight for regularization, crossentropy(·) denotes the cross-entropy loss function, and ||·||_2_ denotes the *L*_2_ regularization term.

Then, the model updates the adaptive dynamic matrix Ã_*D*_ using the following formula:


(20) $$\begin{array}{l} {Ã}_{D}=\left(1-\rho \right){Ã}_{D}+\rho Loss \end{array}$$

here, ρ denotes the learning rate of the model.

### 2.4 Implementation details

The STAFNet model underwent training and evaluation through a five-fold cross-validation protocol (Cheng et al., 2023). In this approach, the dataset is randomly partitioned into five equal-sized subsets. Four of these subsets serve as the training set, while the remaining subset is designated as the test set. For detailed partitioning information, please refer to Table 1. This procedure is iteratively executed five times, ensuring that each subset has the opportunity to function as the test set. To maintain the independence of the training and testing phases, the model is reinitialized following each dataset redivision. The implementation of STAFNet was conducted using PyTorch on an NVIDIA A100 GPU, employing an Adam optimizer with a batch size of 64, an initial learning rate of 0.001, and a weight decay rate of 0.1. Given the use of five-fold cross-validation across all datasets, each fold is trained for 30 epochs, culminating in a total of 150 epochs. The model's final performance metrics are derived by averaging the results across the five folds.

**Table 1:** Datasets overview.

**Dataset**	**Channels**	**Trials**	**Windows**	**Train samples**	**Test samples**
SEED	62	45	1s	121,600	304,00
SEED-IV	62	72	1s	119,316	298,29

### 2.5 Setup of the experiments

To evaluate the classification performance of the STAFNet model, we use four metrics: accuracy, precision, recall, and F1 score, to assess the accuracy and robustness of the multi-class model. The specific formulas are as follows:


(21) $$\begin{array}{c} \text{Accuracy}=\frac{\sum_{i=1}^{N}TP_{i}}{\sum_{i=1}^{N}\left(TP_{i}+FP_{i}+FN_{i}+TN_{i}\right)} \end{array}$$

(22) $$\begin{array}{c} \text{Precision}=\frac{1}{N}\overset{N}{\underset{i=1}{\sum }}\frac{TP_{i}}{TP_{i}+FP_{i}} \end{array}$$

(23) $$\begin{array}{c} \text{Recall}=\frac{1}{N}\overset{N}{\underset{i=1}{\sum }}\frac{TP_{i}}{TP_{i}+FN_{i}} \end{array}$$

(24) $$\begin{array}{c} \text{F}1-\text{score}=\frac{2\text{Precision}\times \text{Recall}}{\text{Precision}+\text{Recall}} \end{array}$$

where *TP*_*i*_, *TN*_*i*_, *FP*_*i*_, and *FN*_*i*_ correspond to the true positives, true negatives, false positives, and false negatives for the *i*-th class, respectively. *N* denotes the total number of classes.

## 3 Results

### 3.1 Emotion recognition results of STAFNet

The STAFNet model exhibited exceptional performance in emotion recognition on both the SEED and SEED-IV datasets, achieving recognition accuracies of 97.89% and 93.64%, respectively. These results indicate that the model retains robust performance when confronted with more complex datasets. As presented in Table 2, the model's performance was assessed from three distinct perspectives: accuracy, recall, and F1 score. On the SEED dataset, the STAFNet model achieved an accuracy of 98.32%, a recall of 97.82%, and an F1 score of 97.75%. In contrast, on the SEED-IV dataset, it achieved an accuracy of 93.84%, a recall of 93.58%, and an F1 score of 93.52%. These findings suggest that the model's performance metrics–accuracy, recall, and F1 score–are comparable across both datasets, underscoring its strong generalization capability. Furthermore, the model sustains high classification performance even in the context of more intricate tasks.

**Table 2:** Performance metrics of the TS-HSTFNet model for different datasets.

**Dataset**	**Classes**	**Subjects**	**Acc(%)**	**Pre(%)**	**Re(%)**	**F1(%)**
SEED	3	15	97.89	98.32	97.82	97.75
SEED-IV	4	15	93.64	93.84	93.58	93.52

To examine the influence of various EEG signal frequency bands on the STAFNet model, we applied a range of bandpass filters during the data preprocessing phase. Specifically, we considered six frequency bands: delta (1–4 Hz), theta (4–8 Hz), alpha (8–13 Hz), beta (13–32 Hz), gamma (32–51 Hz), and an aggregate band encompassing all frequencies (1–51 Hz). The objective was to evaluate the model's ability to classify emotions across these distinct frequency bands.

**Figure 6:**
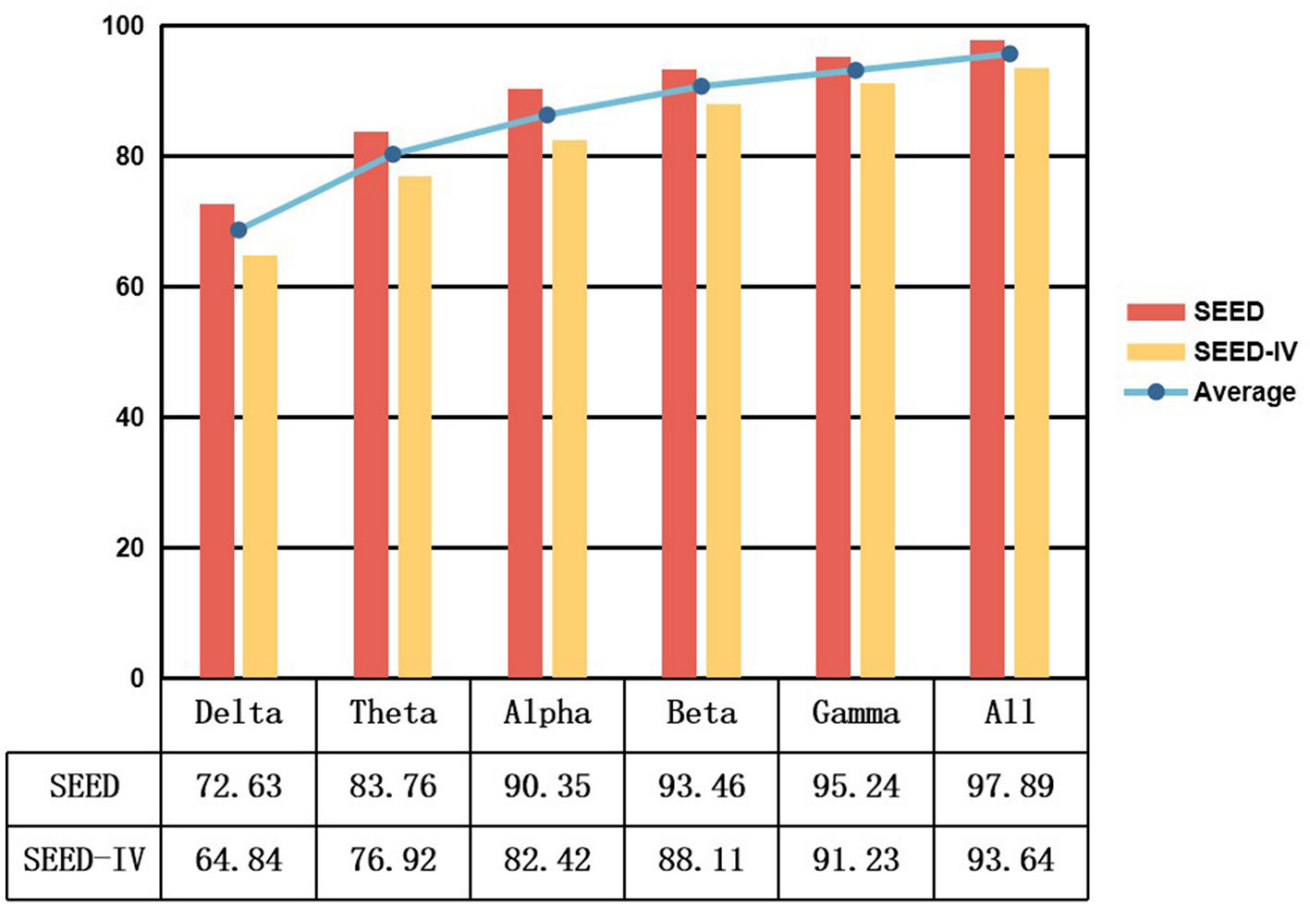
Performance results of the model across different frequency bands.

Figure 6 illustrates the classification results of our model across different frequency bands for the SEED and SEED-IV datasets. The results reveal that the STAFNet model achieves significantly better performance in high-frequency bands, such as Beta and Gamma, compared to low-frequency bands like Delta and Theta. Specifically, the Gamma band demonstrates an increase in accuracy of 22.61% and 26.39% over the Delta band for the SEED and SEED-IV datasets, respectively. This finding indicates that high-frequency bands play a crucial role in enhancing the accuracy of EEG-based emotion recognition tasks. Additionally, utilizing the entire frequency range yields superior performance compared to focusing on individual bands. This suggests that features extracted from various frequency bands are complementary, and their integration contributes to improved classification outcomes in emotion recognition models.

**Figure 7:**
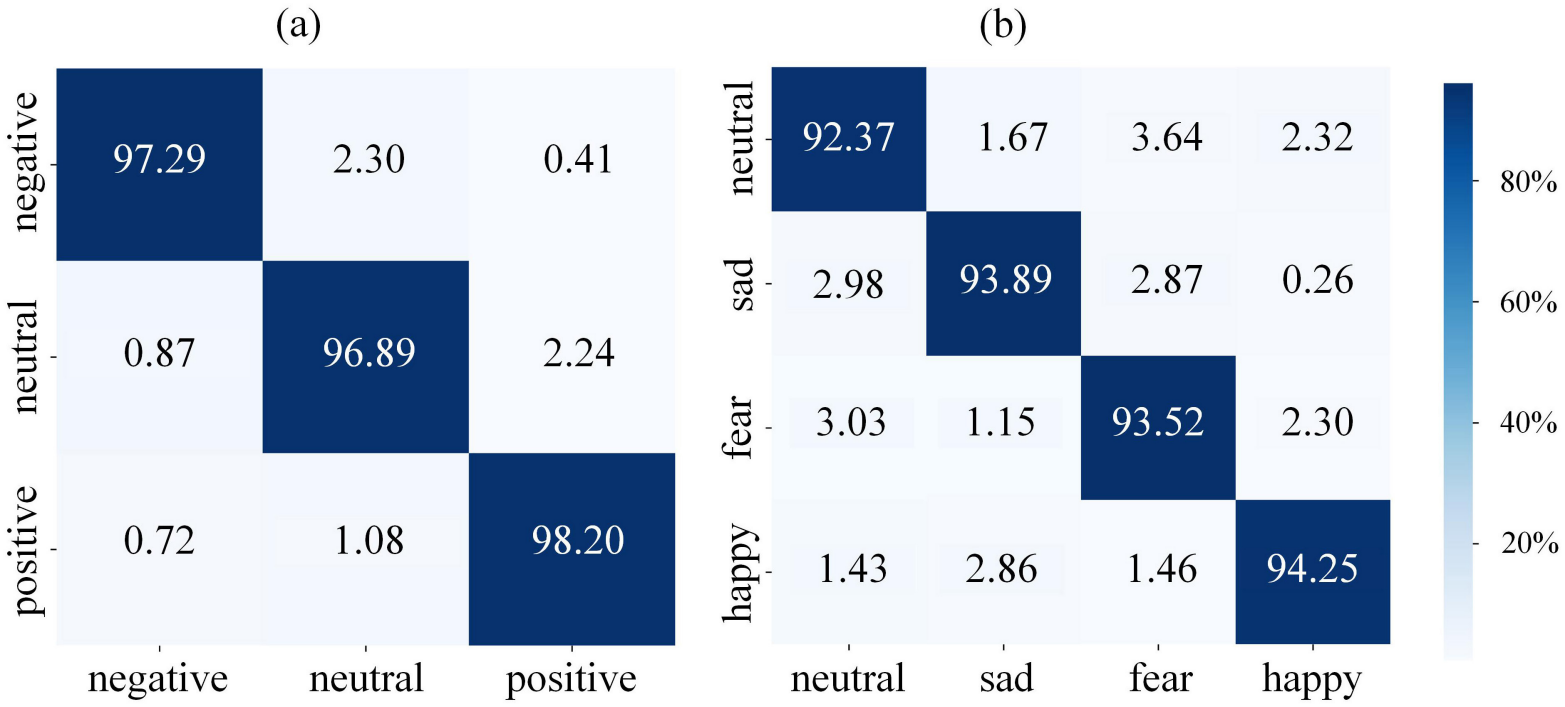
Confusion matrices of the proposed STAFNet model on the **(A)** SEED and **(B)** SEED-IV datasets.

To further validate the overall performance of the STAFNet model on the SEED and SEED-IV datasets, we employed confusion matrices derived from ten-fold cross-validation, as illustrated in Figure 7. The horizontal axis represents the predicted labels, while the vertical axis indicates the true labels. The confusion matrices demonstrate that positive emotions are more readily distinguishable from negative emotions. Specifically, for the SEED dataset, the accuracy of identifying positive emotions is enhanced by 1.31% compared to neutral emotions and by 0.91% compared to negative emotions. Similarly, for the SEED-IV dataset, the accuracy of identifying positive emotions increases by 1.88% relative to neutral emotions, by 0.36% compared to sad emotions, and by 0.73% compared to fearful emotions.

### 3.2 Emotion recognition results of STAFNet

To assess the performance advantages of our model, we compared the STAFNet model against several SOTA methods. A summary of these methods is provided below:

1. 4D-aNN (Xiao et al., 2022): A 4D attention-based neural network has been developed, primarily comprising four convolutional blocks and an attention-based bidirectional long short-term memory (Bi-LSTM) network. Each convolutional block integrates a cascade of spatial and channel attention modules.
2. MDGCN-SRCNN (Bao et al., 2022): We propose a novel model that integrates GCNs and CNNs to extract channel connection features across varying receptive fields, as well as deep abstract features for the differentiation of various emotions.
3. Double way deep neural network (Niu et al., 2023): A brain functional network is constructed based on inter-channel relationships to extract spatial features, while temporal information is extracted from the raw EEG data. The features are ultimately fused through a weighted fusion approach.
4. STGATE (Li J. et al., 2023): A Transformer encoder is utilized to extract time-frequency features, which are then processed through a spatiotemporal graph attention mechanism to perform emotion recognition classification.
5. EEG Conformer (Song et al., 2023): A compact convolutional Transformer is utilized to integrate both local and global features within a cohesive framework for EEG classification.
6. MFFNN (Li M. et al., 2023): A novel multimodal feature fusion neural network model that constructs dual branches to extract both temporal and spatial features.
7. BF-GCN (Li et al., 2024): A graph learning system based on brain cognitive mechanisms and integrated attention mechanisms is proposed. This system employs three types of graph branches to jointly learn emotion recognition patterns from EEG signals.

Table 3 compares the performance of STAFNet against other state-of-the-art methods across two datasets. The results clearly show that our proposed method consistently surpasses advanced techniques in accuracy, underscoring the strong competitiveness of the model introduced in this paper. Several of the evaluated models, such as those in Bao et al. (2022), Song et al. (2023), and Li et al. (2024), primarily focus on either spatial or temporal feature extraction from EEG signals, often neglecting the complementary information shared between these features. The MDGCN-SRCNN model, the top performer among the compared approaches, achieves recognition accuracies of 95.08% on the SEED dataset and 85.52% on SEED-IV. However, while these methods integrate spatiotemporal characteristics, many overlook the critical role of feature fusion strategies, leading to incomplete extraction of essential features and missing complementary information (Xiao et al., 2022; Niu et al., 2023; Li J. et al., 2023; Li M. et al., 2023). The 4D-aNN model proposed in Xiao et al. (2022) comes closest to our model's performance, with accuracies of 96.25% and 86.77% on the SEED and SEED-IV datasets, respectively, but it shows limited capability in leveraging potential associations between multiple features. In contrast, STAFNet not only captures spatiotemporal information from EEG signals but also introduces innovative feature fusion techniques, emphasizing inter-feature correlations and effectively extracting high-level semantic information related to emotions. As a result, our model achieves superior overall performance.

**Table 3:** Average accuracy of different methods on the SEED and SEED-IV datasets.

**References**	**Models**	**SEED**	**SEED-IV**
Xiao et al. (2022)	4D-aNN	96.25	86.77
Bao et al. (2022)	MDGCN-SRCNN	95.08	85.52
Niu et al. (2023)	Double way deep neural network	94.55	78.91
Li J. et al. (2023)	STGATE	90.27	76.43
Song et al. (2023)	EEG Conformer	95.30	-
Li M. et al. (2023)	MFFNN	-	87.32
Li et al. (2024)	BF-GCN	92.72	82.03
This study	AGTFNet	97.89	93.64

### 3.3 Ablation experiment

This paper presents a dual-branch framework from the spatiotemporal perspective of EEG, incorporating a feature fusion mechanism in the model design. To validate the contributions of different components to the STAFNet model, we conducted several ablation studies on the SEED and SEED-IV datasets. The results of these ablation experiments are summarized in Table 4. The models compared include: (1) STAFNet w/o AGCM: where the AGCM is removed; (2) STAFNet w/o TSRM: where the TSRM is omitted; (3) STAFNet w/ PLI: utilizing a static adjacency matrix constructed using the Phase Lag Index (PLI); (4) the MSTFM module replaced by five mainstream fusion methods, including additive fusion (AF), concatenation fusion (CF), spatial attention fusion (SAF), channel attention fusion (CAF), and transformer fusion (TF); and (5) STAFNet: our proposed model.

**Table 4:** Ablation study of the STAFNet model on the SEED and SEED-IV datasets.

**Method**	**SEED**	**SEED-IV**	**Average**
AGTFNet w/o AGCM^a^	83.54	75.65	79.60
AGTFNet w/o TSRM^b^	82.67	76.45	79.56
AGTFNet w/ PLI^c^	90.54	87.87	89.21
AGTFNet w/ AF^d^	84.76	78.39	81.58
AGTFNet w/ CF^d^	84.32	77.56	80.94
AGTFNet w/ SAF^d^	90.23	85.46	87.85
AGTFNet w/ CAF^d^	91.56	86.58	89.07
AGTFNet w/ TF^d^	93.78	89.43	91.61
AGTFNet	97.89	93.64	95.77

^a^Without the AGCM in AGTFNet.

^b^Without the TSRM in AGTFNet.

^c^Dynamic adjacency matrix replaced with PLI.

^d^MSTFM replaced with five fusion strategies.

The ablation study results shown in Table 4 indicate that the STAFNet model significantly outperforms other models on the SEED and SEED-IV datasets. Specifically, compared to the single-branch feature extraction methods STAFNet w/o AGCM and STAFNet w/o TSRM, our model achieves an average accuracy improvement of 16.17% and 16.21%, respectively, demonstrating the substantial advantage of the dual-branch spatiotemporal framework in feature extraction. To investigate the contribution of adaptive dynamic graph convolution to the model, we replaced the adaptive adjacency matrix with the PLI adjacency matrix, resulting in an overall average recognition accuracy increase of 6.56%.

Furthermore, the comparison of five mainstream fusion strategies demonstrates that traditional fusion methods have limited capability in capturing complex relationships between multiple features. Specifically, the basic fusion strategies, AF and CF, show average accuracy improvements of 1.98% and 1.34% over STAFNet w/o AGCM, and 2.02% and 1.38% over STAFNet w/o TSRM, respectively. However, these improvements are not significant, indicating that AF and CF have limitations in leveraging the complementary information between spatial and temporal features and capturing the complex relationships among features. In contrast, the attention-based fusion strategies SAF and CAF enhance the model's performance to some extent by dynamically adjusting feature weights and focusing on key areas with strong spatiotemporal feature correlations. However, these attention-based fusion strategies may tend to overly focus on local features while neglecting more global contextual information, which can constrain the overall performance of the model. Among the five fusion strategies, the TF strategy shows the greatest improvement in model performance, achieving accuracies of 93.78% and 89.43% on the SEED and SEED-IV datasets, respectively. This result highlights the efficiency of the TF strategy in capturing long-range dependencies and understanding broad context. However, it has limitations in handling more nuanced interactions between local features and global context. To address this limitation, the MSTFM proposed in this paper provides an innovative enhancement over TF by facilitating the interaction between temporal and spatial features, making it easier to capture the potential dependencies between features. In summary, by combining the advantages of the AGCM, TSRM, and MSTFM, we have developed the final model, STAFNet, which demonstrates significantly improved performance compared to models that focus on single features or other fusion strategies.

**Figure 8:**
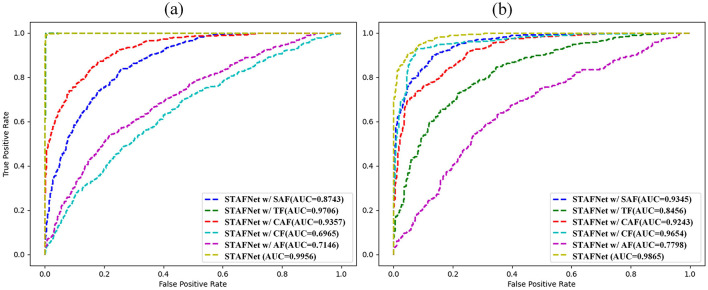
Comparison of AUC curves of five ablation experimental studies on different datasets. **(A)** SEED. **(B)** SEED-IV.

Additionally, to further investigate the impact of different fusion strategies on model performance, we analyzed the ROC curves of STAFNet, STAFNet w/ AF (AF), STAFNet w/ CF (CF), STAFNet w/ SAF (SAF), STAFNet w/ SCF (SCF), and STAFNet w/ TF (TF), as shown in Figure 8. On the SEED dataset, our model outperformed others, achieving a maximum AUC of 0.9956, with respective improvements of 0.281 (AF), 0.2991 (CF), 0.1213 (SAF), 0.0599 (SCF), and 0.025 (TF). Similarly, on the SEED-IV dataset, STAFNet attained an AUC of 0.9865, surpassing AF, CF, SAF, SCF, and TF by 0.2067, 0.0211, 0.052, 0.0622, and 0.1409, respectively, demonstrating its superior performance. In summary, the MSTFM module's fusion approach exhibits clear advantages over other fusion strategies.

## 4 Discussion

### 4.1 The impact of GCN layer depth on model performance

**Figure 9:**
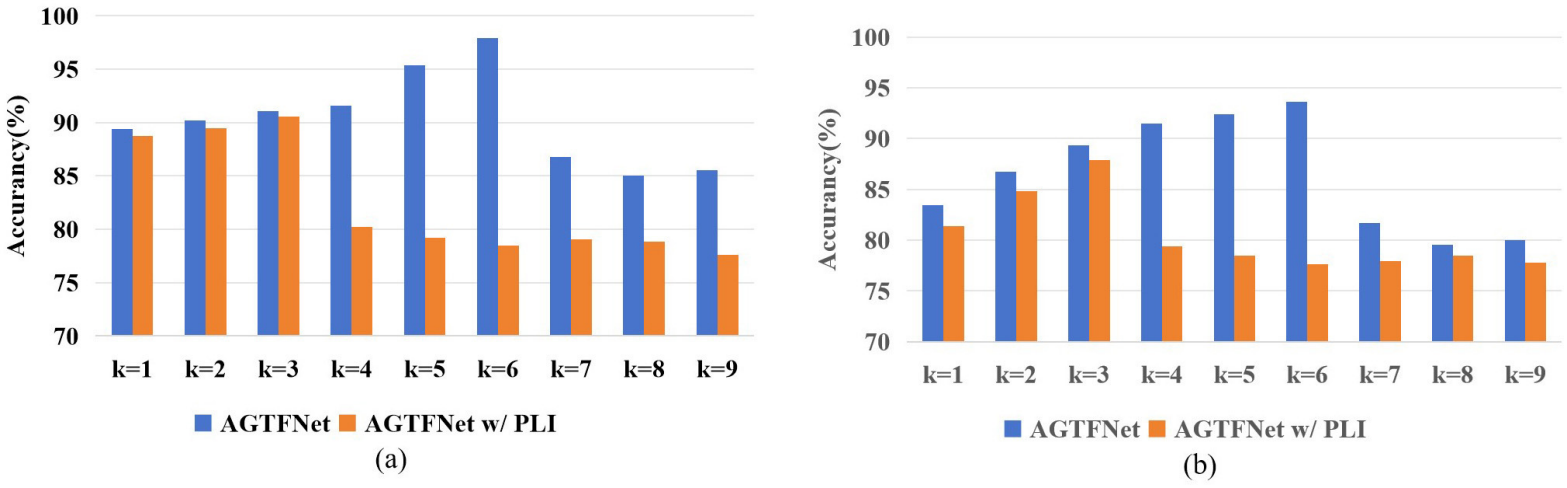
**(A)** The effect of the number of layers k on the performance of STAFNet w/PLI and STAFNet models on the SEED dataset; **(B)** The effect of the number of layers k on the performance of STAFNet w/PLI and STAFNet models on the SEED-IV dataset.

GCN updates node representations by considering each node's features and aggregating information from all its neighbors. Given the complex spatial dependencies in the brain, deeper GCN network structures are needed to obtain richer node feature representations. However, as the network depth increases, the learned node features tend to become more homogeneous, which can lead to decreased classification performance. Therefore, we will investigate the impact of the number of GCN layers *k* on model performance in STAFNet w/ PLI (based on PLI) and STAFNet (based on adaptive adjacency matrices) to demonstrate that the STAFNet model can capture deeper spatial features and mitigate the over-smoothing problem, as illustrated in Figure 9.

According to the results shown in Figure 9, the STAFNet model exhibits superior performance compared to the STAFNet w/ PLI model on both the SEED and SEED-IV datasets, with overall performance improvements of 7.35% and 5.77%, respectively. The STAFNet model achieves its best performance at *k* = 6, whereas the STAFNet w/ PLI model reaches its highest accuracy at *k* = 3. Additionally, for smaller network depths, model performance improves as the number of GCN layers increases. However, as the network depth grows beyond a certain point, the key information in node features tends to become homogenized, leading to the over-smoothing problem. Therefore, the results indicate that the AGCM, based on adaptive adjacency matrices, helps mitigate the over-smoothing issue and capture deeper spatial dependencies.

### 4.2 t-SNE

**Figure 10:**
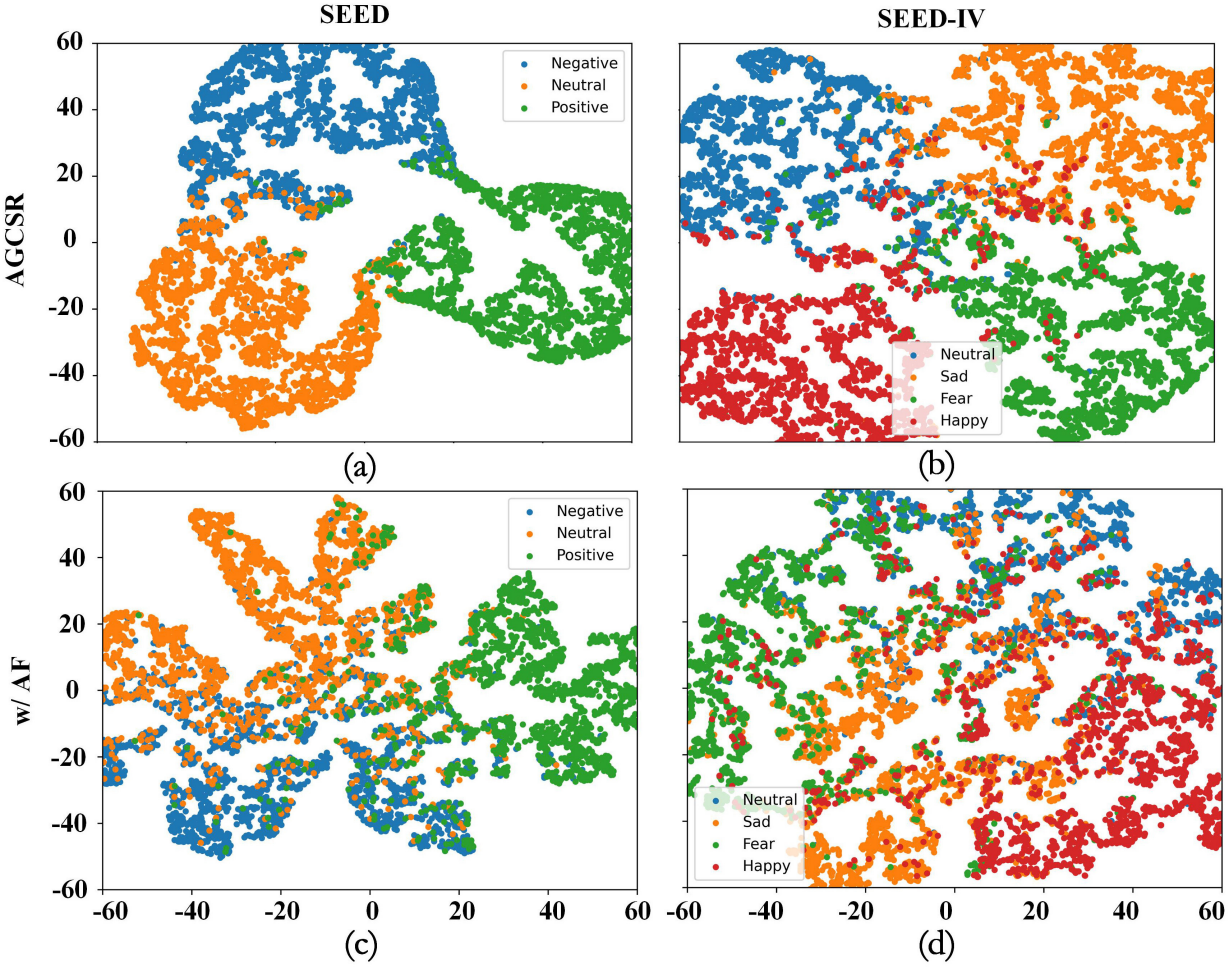
t-SNE visualization. Different colors represent different emotion categories. **(A)** Feature distribution of the STAFNet model using AGCSR on the SEED dataset. **(B)** Feature distribution of the STAFNet model using AGCSR on the SEED-IV dataset. **(C)** Feature distribution of the STAFNet model using the AF method on the SEED dataset. **(D)** Feature distribution of the STAFNet model using the AF method on the SEED-IV dataset.

To visually demonstrate the classification performance and effectiveness of MSTFM, we used t-SNE to visualize the high-dimensional feature space distributions of the SEED and SEED-IV emotion recognition tasks for the STAFNet model and the STAFNet w/ AF model (where MSTFM is replaced with the AF module). The results are shown in Figure 10. The visualization reveals that the STAFNet w/ AF model has relatively close inter-cluster distances for different emotion categories, leading to noticeable overlap between categories. In contrast, the STAFNet model shows larger inter-cluster distances and smaller intra-cluster distances, resulting in clearer boundaries between feature clusters for different emotion types. Specifically, in the SEED dataset, the STAFNet model shows that the feature clusters for positive emotions have smaller intra-cluster distances and less overlap with neutral and negative emotions, maximizing the distinction between positive and other emotions, which is consistent with the results obtained from the confusion matrix. In the SEED-IV dataset, the cohesion of feature clusters for sadness and positive emotions is significantly better compared to fear and neutral emotions. These observations are closely related to the performance of the STAFNet model in emotion recognition tasks, confirming the crucial role of MSTFM in enhancing the model's classification accuracy.

## 5 Conclusions

In this paper, we introduce STAFNet, a novel spatiotemporal feature fusion network designed for emotion recognition by effectively integrating complementary information from both spatial and temporal features. The AGCM dynamically captures brain connectivity patterns, extracting critical spatial features from multiple nodes. Simultaneously, the TSRM evaluates the global importance of different time segments within each EEG sample, producing more representative temporal features. These spatial and temporal features are then fused through the MSTFM, enabling the model to capture invariant feature representations and boost performance. Extensive experiments on the SEED and SEED-IV datasets demonstrate that STAFNet outperforms several SOTA models, as well as in ablation studies. Our results validate the efficacy of STAFNet in EEG-based emotion recognition, showing notable improvements in extracting informative features from EEG signals and enhancing recognition performance. This work emphasizes the importance of jointly considering spatiotemporal features for emotion recognition. Future work will explore constructing global dynamic graphs and regional functional maps based on consistent activation patterns between emotions and specific brain regions. Additionally, while this study highlights model generalizability, future research should incorporate subject-independent experiments.

## Data Availability

The datasets presented in this study can be found in online repositories. The names of the repository/repositories and accession number(s) can be found below: SEED Dataset (https://bcmi.sjtu.edu.cn/~seed/seed.html) and SEED-IV Dataset (https://bcmi.sjtu.edu.cn/~seed/seed-iv.html).
